# Subnitrate of Bismuth

**Published:** 1854-12

**Authors:** 


Subnitrate of Bismuth.—M. Caby recommends strongly the local application of bismuth, by means of the speculum and lint, on the neck and mouth of the uterus, and on the vagina, as the speculum is being withdrawn. There is no pain. The application should be made daily. Before using the bismuth a water injection may be used, to clear away the discharge from the membrane.—*Bull. Gén. de Thér.*

